# Cervical Cancer Screening Outcomes Among a Sample of Low-Income Uninsured Women: A Program-Based Study

**DOI:** 10.1089/whr.2021.0091

**Published:** 2022-01-31

**Authors:** Samson Olowolaju, Morgan Kassabian, Marvellous A. Akinlotan, Anna Lichorad, Robert Pope, Brandon Williamson, Scott Horel, Jane N. Bolin

**Affiliations:** ^1^ Department of Health Policy and Management, Texas A&M University School of Public Health, TAMU, College Station, Texas, USA.; ^2^ Texas A&M College of Nursing, Texas A&M University, College Station, Texas, USA.; ^3^ Department of Primary Care and Population Health, Texas A&M College of Medicine, Bryan, Texas, USA.

**Keywords:** cervical cancer, outcomes, Pap test, HPV, colposcopy

## Abstract

***Background:*** Most studies examining cervical cancer screening outcomes have focused on either an age-specific diagnosis and outcomes of abnormal smears or frequency of abnormal outcomes among a sample of insured women. Thus, it is unclear what the distribution outcomes would be when other sociodemographic characteristics are considered. This study examines the variation in cervical cancer screening outcomes and sociodemographic characteristics (patients' age, marital status, race/ethnicity, rurality, and Papanicolaou [Pap] test screening history) within a sample of low-income and uninsured women.

***Materials and Methods:*** Our grant-funded program provided 751 Pap tests, 577 human papillomavirus (HPV) tests, and 262 colposcopies to 841 women between 2013 and 2019. Observed outcomes for each procedure type were cross-tabulated by patients' sociodemographic characteristics. Chi-squared and Fisher's exact tests were used to test the independence of screening outcomes and sociodemographic characteristics.

***Results:*** The overall positivity rate was 7.2% for Pap tests (*n* = 54/751), 3.6% for HPV tests (*n* = 21/577), and 44.7% for colposcopies (*n* = 117/262). Significance tests suggested that the Pap test and colposcopy outcomes we observed were independent of sociodemographic characteristics in all but one instance—Pap test outcomes were not independent of patient age (*p* = 0.009). Moreover, the Pap test positivity rate increased with patient age.

***Conclusions:*** Our findings support recommendations to discontinue screening for women older than 65 years at low risk for cervical cancer. Our ability to identify an association between cervical screening outcomes and other sociodemographic characteristics may have been limited by our small sample size. This highlights an important barrier to studying health outcomes within low-income and uninsured populations, which are often missing in larger research data sets (*e.g.*, claims).

## Introduction

Although cervical cancer is one of the most preventable types of cancer in developed countries, its prevention requires protection against human papillomavirus (HPV) infection through vaccination and/or regular testing to identify abnormal cells early enough for appropriate treatment.^1^ HPV testing, routine Papanicolaou (Pap) tests, and colposcopy are procedures that are used alone or in combination to screen for and diagnose cervical cancer disease.

While the most important risk factor for developing cervical cancer is an infection with HPV,^2^ Pap tests are used to screen for cervical cancer by identifying whether there are cells from the cervix that appear cancerous or precancerous.^3^ Colposcopy is a diagnostic test for cervical cancer that is used to make a definitive diagnosis following abnormal Pap test findings.^4^ Owing to these measures, as well as HPV vaccinations which have been available to adolescents and young adults since 2006,^5^ the United States has been able to reduce the incidence and mortality rate for cervical cancer by 75% since the 1940s.^1^

Despite an overall decline in the number of new cases and deaths from cervical cancer in the United States, disparities exist among population groups in the rate of decline in the incidence of the disease.^6,7^ There is evidence that more than 60% of invasive cervical cancers occur in underserved populations in the United States.^1^ These underserved populations include low-income women, women living in rural areas, ethnic minorities, and undocumented immigrants without health insurance.^1^ Members of these groups experience a higher risk of cervical cancer due to lack of access to screening, and a lower likelihood of utilizing follow-up care.^1,8,9^

Literature suggests that socioeconomic status is an important determinant of a woman's likelihood of obtaining cervical cancer screening, diagnosis, and treatment.^10^ In light of this, it may not be surprising that rural areas, which are generally characterized by poorer less educated populations,^11^ experience significantly higher rates of cervical cancer incidence and mortality than urban areas.^12,13^ However, research has shown that the negative relationship between rurality and cervical cancer incidence remains significant even after local socioeconomic status variables and primary care density are accounted for.^14^ This suggests that both socioeconomic factors and rurality are important variables to include in studies concerning cervical cancer risk.

Most published studies examining the outcomes of cervical cancer screening have focused on either an age-specific diagnosis and outcomes of abnormal routine smears or frequency of abnormal outcomes among a sample of women with health insurance. As a result, it is unclear what the prevalence of abnormal outcomes would be when other sociodemographic characteristics are considered. Examining the prevalence of abnormal outcomes for Pap tests, based on sociodemographic characteristics among a group of low-income uninsured women may offer better insight to the study of the subject.

To that end, the purpose of this article was to examine the variation in cervical cancer screening outcomes by sociodemographic characteristics among a sample of low-income uninsured women participating in a grant-funded program. These characteristics include patients' age, marital status, race/ethnicity, rurality, and Pap test screening history.

## Materials and Methods

### Data source

The study sample for this article consisted of uninsured women who had received a routine Pap test and/or a follow-up colposcopy. All patients received services as part of a grant-funded project implemented at a university-affiliated family medicine clinic in central Texas. Since March 2013, the program has made it possible for low-income uninsured women to access breast, cervical, and colorectal cancer screenings in counties in and around the Brazos Valley region of Texas. The data set was provided under Texas A&M Institutional Review Board protocol 2013-0885D related to grant awards PP130090 and PP170037 from the Cancer Prevention and Research Institute of Texas.

### Study sample

A total of 841 women received cervical cancer tests between 2013 and 2019. To analyze clinical outcomes for cervical screening among our study sample, women who had Pap test, diagnostic colposcopy, or both within the project period were selected.

Among the 841 women, a total number of 751 Pap tests (Table 1), 577 HPV tests (Table 2), and 262 diagnostic colposcopies (Table 3) were conducted. Notably, not all women who obtained a Pap test through our program and received abnormal Pap results chose to accept our recommendation to follow up with colposcopy. Additionally, not all women who received colposcopy services through our program also received a Pap test from us, as colposcopy referrals from outside clinics were accepted for women who met the grant's income and residence requirements.

**Table 1.** Routine Papanicolaou Test Outcomes by Sociodemographic Characteristics (2013–2019)

**Characteristic**	**Pap test screening outcomes**						
	**Total *n* = 751 (100.0%)**	**Negative *n* = 697 (92.8%)** ^a^	**Positive**				***p*** ^b^
			**ASCUS *n* = 34 (4.5%)** ^a^	**LGSIL *n* = 14 (1.9%)** ^a^	**HGSIL *n* = 5 (0.7%)** ^a^	**U/S *n* = 1 (0.1%)** ^a^	
Age, years							0.009
20–24	22	17 (77.3%)	2 (9.1%)	3 (13.6%)	—	—	
25–29	44	40 (90.9%)	2 (4.5%)	2 (4.5%)	—	—	
30–39	148	135 (91.2%)	9 (6.1%)	2 (1.4%)	2 (1.4%)	—	
40–49	236	219 (92.8%)	12 (5.1%)	1 (0.4%)	3 (1.3%)	1 (0.4%)	
50–59	238	224 (94.1%)	8 (3.4%)	6 (2.5%)	—	—	
60–69	63	62 (98.4%)	1 (1.6%)	—	—	—	
Marital status							0.8
Married	245	225 (91.8%)	15 (6.1%)	3 (1.2%)	1 (0.4%)	1 (0.4%)	
Single	314	292 (93.0%)	11 (3.5%)	8 (2.5%)	3 (1.0%)	—	
Widow	2	2 (100.0%)	—	—	—	—	
Missing	190	178	8	3	1	0	
Race/ethnicity							0.6
White	239	222 (92.9%)	10 (4.2%)	4 (1.7%)	3 (1.3%)	—	
Black	157	149 (94.9%)	6 (3.8%)	1 (0.6%)	1 (0.6%)	—	
Hispanic	313	286 (91.4%)	16 (5.1%)	9 (2.9%)	1 (0.3%)	1 (0.3%)	
Other	4	4 (100.0%)	—	—	—	—	
Missing	38	36	2	—	*—*	*—*	
Rurality							0.9
Urban	583	540 (92.6%)	26 (4.5%)	12 (2.1%)	4 (0.7%)	1 (0.0%)	
Large rural	34	30 (88.2%)	3 (8.8%)	1 (2.9%)	—	—	
Small rural	64	61 (95.3%)	3 (4.7%)	—	—	—	
Isolated	63	59 (93.7%)	2 (3.2%)	1 (1.6%)	1 (1.6%)	—	
Missing	7	7	—	—	—	—	
Ever had Pap test							N/A^c^
Yes	513	474 (92.4%)	24 (4.7%)	13 (2.5%)	2 (0.4%)	—	
No	16	16 (100%)	—	—	—	—	
Don't know	62	55 (88.7%)	4 (6.5%)	1 (1.6%)	1 (1.6%)	1 (2%)	
Missing	160	152	6	—	2	*—*	

^a^ Percentages represent the cell's value divided by the total number of tests for that row. Percentages are rounded and may not sum up to 100% across rows.

^b^ *p*-Values represent the probability of a nonrandom association between a sample characteristic and any positive Pap test screening outcome.

^c^ “Ever Had Pap Test” variable not included in the *F*-test, as to be consistent with the *F*-test performed on colposcopy outcomes.

ASCUS, atypical squamous cells of undetermined significance; HGSIL, high-grade squamous intraepithelial lesion; LGSIL, low-grade squamous intraepithelial lesion; N/A, not applicable; Pap test, Papanicolaou test; U/S, unsatisfactory sample.

**Table 2.** Human Papillomavirus Test Outcomes by Sociodemographic Characteristics (2013–2019)

**Characteristic**	**HPV test outcomes**		
	**Overall *n* = 577 (100.0%)**	**Negative *n* = 556 (96.4%)**	**Positive *n* = 21 (3.6%)**
Age, years			
20–24	13	12 (92.3%)	1 (7.7%)
25–29	27	26 (96.3%)	1 (3.7%)
30–39	116	113 (97.4%)	3 (2.6%)
40–49	177	168 (94.9%)	9 (5.1%)
50–59	195	189 (96.9%)	6 (3.1%)
60–69	49	48 (98.0%)	1 (2.0%)
Marital status			
Married	184	176 (95.7%)	8 (4.3%)
Single	213	203 (95.3%)	10 (4.7%)
Widow	1	1 (100.0%)	—
Missing	179	176	3
Race/ethnicity			
White	190	178 (93.7%)	12 (6.3%)
Black	110	106 (96.4%)	4 (3.6%)
Hispanic	244	239 (98.0%)	5 (2.0%)
Other	4	4 (100%)	—
Missing	29	29	—
Rurality			
Urban	448	432 (96.4%)	16 (3.6%)
Large rural	30	28 (93.3%)	2 (6.7%)
Small rural	48	46 (95.8%)	2 (4.2%)
Isolated	45	44 (97.8%)	1 (2.2%)
Missing	6	6	—
Ever had Pap test			
Yes	388	371 (95.6%)	17 (4.4%)
No	12	12 (100.0%)	—
Don't know	45	43 (95.6%)	2 (4.4%)
Missing	132	130	2

HPV, human papillomavirus.

**Table 3.** Colposcopy Outcomes by Sociodemographic Characteristics (2013–2019)

**Characteristic**	**Colposcopy screening outcomes**						
	**Overall *n* = 262 (100.0%)**	**Negative (false positive) *n* = 145 (55.3%)** ^a^	**Positive**				***p*** ^b,c^
			**CIN1 *n* = 62 (23.7%)** ^a^	**CIN2 *n* = 27 (10.3%)** ^a^	**CIN3 *n* = 27 (10.3%)** ^a^	**Invasive *n* = 1 (0.4%)** ^a^	
Age, years							0.3
20–24	22	10 (45.5%)	4 (18.2%)	4 (18.2%)	4 (18.2%)	—	
25–29	53	31 (58.5%)	16 (30.2%)	5 (9.4%)	1 (1.9%)	—	
30–39	101	49 (48.5%)	27 (26.7%)	10 (9.9%)	14 (13.9%)	1 (1.0%)	
40–49	54	31 (57.4%)	12 (22.2%)	7 (13.0%)	4 (7.4%)	—	
50–59	23	15 (65.2%)	3 (13.0%)	1 (4.3%)	4 (17.4%)	—	
60–69	9	9 (100.0%)	—	—	—	—	
Marital status							0.3
Married	69	44 (63.8%)	14 (20.3%)	4 (5.8%)	7 (10.1%)	—	
Single	154	83 (53.9%)	35 (22.7%)	20 (13.0%)	16 (10.4%)	—	
Missing	39	18	13	3	4	1	
Race/ethnicity							0.2
White	54	27 (50.0%)	12 (22.2%)	6 (11.1%)	8 (14.8%)	1 (1.9%)	
Black	40	21 (52.5%)	7 (17.5%)	10 (25.0%)	2 (5.0%)	—	
Hispanic	153	90 (58.8%)	40 (26.1%)	10 (6.5%)	13 (8.5%)	—	
Other	1	1 (100.0%)	—	—	—	—	
Missing	14	6	3	1	4	—	
Rurality							0.06
Urban	191	106 (55.5%)	41 (21.5%)	20 (10.5%)	23 (12.0%)	1 (0.5%)	
Large rural	25	8 (32.0%)	10 (40.0%)	5 (20.0%)	2 (8.0%)	—	
Small rural	26	17 (65.4%)	7 (26.9%)	1 (3.8%)	1 (3.8%)	—	
Isolated	20	14 (70%)	4 (20%)	1 (5.0%)	1 (5.0%)	—	

^a^ Percentages represent the cell's value divided by the total number of tests for that row. Percentages are rounded and may not sum up to 100% across rows.

^b^ CIN2 and CIN3 were combined when conducting significance tests due to small sample size. Similarly, the single test with an invasive outcome was excluded.

^c^ *p*-Values represent the probability of a nonrandom association between a sample characteristic and the distribution of grouped colposcopy screening outcomes (false positive, CIN1, CIN2/CIN3).

CIN, cervical intraepithelial neoplasia.

### Outcome variables

Outcomes of routine cervical screenings were categorized as negative, atypical squamous cells of undetermined significance (ASCUS), low-grade squamous intraepithelial lesion (LGSIL), high-grade squamous intraepithelial lesion (HGSIL), unsatisfactory samples, and HPV^+^ (Tables 1 and 2). A patient was categorized to be HPV^+^ if such patient's HPV screening test was positive for either HPV 16 or HPV 18. This follows the American Society for Colposcopy and Cervical Pathology 2012 Algorithms and guidelines for managing abnormal cervical cancer screening tests and cancer precursors.^15^

Outcomes of colposcopies, following abnormal Pap tests, were classified as follows: negative (false positive), cervical intraepithelial neoplasia (CIN)1, CIN2, CIN3, and invasive cancer (Table 3). In cases where multiple outcomes were recorded for a patient with multiple colposcopy procedures, the most severe outcome was reported. If a patient had a negative outcome from a colposcopy screening, a false-positive outcome was assigned for such patient. These classifications are consistent with the literature.^16^

### Independent variables

The sociodemographic variables included in our model included age, marital status, race/ethnicity, rurality, and Pap test screening history.

Age was observed as a categorical variable, which grouped patients' age into 5-year cohorts (20–24, 25–29, 30–39, 40–49, 50–59, 60–69).

Patient-reported marital status was categorized as “Married,” “Single,” or “Widowed.” Patients were given the option of skipping this question, and nonresponses were marked as “Missing.”

Similarly, patient-reported race/ethnicity was observed as “White,” “Black,” “Hispanic,” “Others,” or “Missing,” where the “Others” category included patients who identified as Asian, American Indian/Alaskan Natives, and Native Hawaiian/Pacific Islander.

Rurality was defined using the four categories of Rural–Urban Commuting Areas (RUCAs) classification, that is, urban, large rural, small rural, and isolated.

Finally, the variable, “Ever Had Pap Test,” captures patients' reported prior cervical cancer screening history, allowing for “Yes,” “No,” and “Don't Know” responses, as well as “Missing.”

### Analysis

Frequencies for clinical outcomes for routine Pap tests (Table 1), HPV tests (Table 2), and colposcopies (Table 3) were cross-tabulated to show their distribution across sociodemographic characteristics. In addition, chi-squared test statistics were used to test for the independency of Pap test and colposcopy outcomes from sociodemographic characteristics. As is commonly done when working with limited sample sizes, Fisher's exact tests were used in lieu of chi-squared test statistics when expected cell counts were <5.

Since the occurrence of any positive outcome was rare, all positive Pap outcomes (cytological outcomes) were grouped as “abnormal,” and negative outcomes were grouped as “normal” to have a large enough sample to test our hypothesis. Similarly, when examining positive colposcopy outcomes (histological outcomes), CIN2 and CIN3 outcomes were combined in testing the stated hypothesis. Following advice from clinical faculty, the single colposcopy test that resulted in an “invasive” outcome was excluded—this outcome was deemed too dissimilar to group with CIN2 and CIN3, yet it was too rare an outcome to include in the analysis.

## Results

### Routine Pap tests

Table 1 displays the distribution of cervical cancer screening outcomes by observed sociodemographic characteristics. Among 751 women who had routine Pap tests over the 6-year period, most (92.8%) had a normal/negative cervical screening outcome. Across all patients screened in the 6-year period, ASCUS was diagnosed at the rate of 4.5 per 100, compared with 1.9 per 100 for LGSIL, and 0.7 per 100 for HGSIL. Only one sample was unsatisfactory.

Women in age groups 50–59 and 40–49 had the highest routine cervical cancer screening utilization—together, these two age groups accounted for nearly two-thirds of the total Pap tests performed during the observation period. The positivity rate appeared to decrease with increasing patient age. The highest recorded positivity rate, about 23%, was observed among women aged 20–24 years, whereas the lowest rates of abnormalities were observed among the two highest age groups (50–59 and 60–69 years).

Among women who were screened and indicated their marital status, more than half were single (*n* = 314). The rate of abnormality among married women was slightly higher than the rate observed among single women, at about 8.2% and 7.0%, respectively.

In terms of race/ethnicity, the rate for abnormal Pap results was highest among Hispanics (8.6%), followed by Whites (7.1%) and then Blacks (5.1%). Regarding rurality, 7.4% of women residing in an urban area had an abnormal outcome, 11.8% in large rural communities, 4.7% in small rural areas, and 6.3% in isolated communities.

Among women who had routine Pap tests during this period and who responded to the question on previous routine Pap testing, 86.8% had previously had Pap test screening, 10.5% were not sure if they had a Pap test in the past, and only 2.7% had never had a Pap test.

### HPV tests

Table 2 reports HPV testing outcomes by sociodemographic characteristics. Among patients who received a Pap test and HPV test within the same visit (*n* = 577), 3.6% had an abnormal/positive outcome, whereas a majority (96.4%) had a normal/negative result. Consistent with cytological outcomes, age group 20–24 had the highest prevalence rate of HPV (7.9%) and age group 60–69 had the lowest HPV prevalence rate (2.0%). For the remaining five age groups, we observed positivity rates that fell between these high and low values (spanning 3.1.%–5.1%), but that did not follow a consistent directional pattern.

Patients who reported single as their marital status had a higher observed prevalence rate of HPV infection (4.7%) than married patients, who had an observed rate of 4.3%. Regarding race/ethnicity, HPV infection was most prevalent among Whites (6.3%), followed by Blacks (3.6%) and Hispanics (2.0%). Patients who resided in large rural areas had the highest rate of HPV infection (6.7%), small rural residents had a rate of 4.2%, and patients from urban and isolated areas had prevalence rates of 3.6% and 2.2%, respectively.

### Colposcopies

Table 3 presents the prevalence of CIN and invasive cancer outcomes from colposcopy tests as a rate per 100 women. Across all women who had a colposcopy test, the prevalence of CIN1 was 23.7 per 100, with rates of 10.3 per 100 for CIN2, 10.3 per 100 for CIN3, and <1% of invasive cancer. Negative colposcopy test results are also referred to as “false positives” since they indicate that cervical cells are normal, rather than abnormal as suggested by the initial Pap test that would have triggered referral for colposcopy. We observed a false positive rate of 55.3 per 100 women. The highest rate of CIN1 (30.2%) was observed for women in the 25–29 age group, whereas the rate of CIN2 (18.2%) and CIN3 (18.2%) peaked in the 20–24 age group.

Histological outcomes were also examined by marital status. Consistent with cytological outcomes, the positivity rate was higher for single women (46.1%) than for married women (36.2%). Among single women, 22.7% received a colposcopy outcome of CIN1, where 13.0% and 10.4% received outcomes of CIN2 and CIN3, respectively. When race/ethnicity was considered, the highest rate of CIN1 (26.1%) was observed among women of Hispanic origin, whereas the rates of CIN2 (25.0%) and CIN3 (14.8%) peaked for Black race and White race, respectively.

Table 3 also shows the distribution of these outcomes with respect to rurality. While the observed rates of CIN1 (40.0%) and CIN2 (20.0%) were higher among women residing in large rural areas, the rate of CIN3 (12.0%) peaked among women who reside in urban areas.

### Significance testing

Results from the chi-squared and Fisher's exact tests showed that the distribution of cytological outcomes (negative/normal test result or any positive finding including ASCUS, LGSIL, or HGSIL) was not independent of age group (*p* = 0.009). Significance tests did suggest, however, that the cytological outcomes we observed were independent of marital status (*p* = 0.8), race/ethnicity (*p* = 0.6), and rurality (*p* = 0.9).

When examining histological outcomes (false positive, CIN1, and CIN2/3), the significance tests performed indicated that the distribution of outcomes was independent of all the tested sociodemographic characteristics: age group (*p* = 0.3), marital status (*p* = 0.3), race/ethnicity (*p* = 0.2), and rurality (*p* = 0.06).

## Discussion

The results of our analysis showed a decline in the rate of positive cytological outcomes with increased age, similar to that of histological trends. Thus, our study confirmed and supported the recommendations of the U.S. Preventive Services Task Force to discontinue screening for women older than 65 years at low risk for cervical cancer.^17^ As shown in Table 1, only 1.6% (*n* = 1) of women aged 60 years and older had a positive cytological outcome, whereas 100% of women in this age group who presented for histological screening had a false-positive screening outcome. Evidence from prior studies supports our findings.^18^

Our sample analysis shows an overall prevalence rate for ASCUS (4.5%), LGSIL (1.9%), and HGSIL (0.7%). These findings are similar to that of a College of American Pathologists Laboratory Improvement Program study of 323 laboratories that found a median rate of ASCUS (4.5%), LGSIL (1.6%), and HGSIL (0.5%).^19^

Cervical cancer can often be avoided by having regular Pap and HPV testing to find precancerous tissue and treat it—before it becomes cancer. While preventative screening is critical in terms of saving lives, the financial burden of cervical cancer is also worth examining. Cervical cancer treatment can cost between $30,700 and $52,600 per case (2010 USD), excluding the cost of post-Pap follow-up procedures.^20^

Screening programs, such as the one described in this article, assist in reducing the cost of expensive treatment and can, therefore, be of particular value to low-income and uninsured women. Given the number of abnormal screening results detected (54 of 751 Pap tests, Table 1), our program helped to avoid unneeded spending from cases that may have progressed to invasive cancer if left undetected. Approximately, this program may have saved society between $1,657,800 and $2,840,400 (2010 USD) in total medical costs of treating 54 cases of the disease.

While our sample of 841 low-income uninsured women could be considered large in the context of studies focused on difficult-to-reach priority populations, it is small in the context of all cross-sectional research studies, which mostly rely on national data that include insured individuals. As a result, the relatively small sample size available for this study necessitated several methodological limitations. For example, while it would have been ideal to regress sociodemographic characteristics on observed cervical cancer screening outcomes as to make inferences about the relationship between these variables in the population, in most cases, our small sample size limited us to running Fisher's exact tests.

This, in effect, has limited the relevance of our conclusions to our sample, although it does not preclude them from being instructive in guiding future larger scale studies. In addition, our small sample size necessitated that outcomes be grouped when conducting significance tests. Because the occurrence of any positive outcome was rare, categories of positive outcomes had to be grouped together (all positive Pap outcomes and colposcopy outcomes of CIN2 and CIN3) to achieve a sample size large enough to complete the test. This was not done, however, without consultation with an experienced obstetrics and gynecology physician, who confirmed that these grouping are clinically relevant.

An additional limitation of our study worth noting is that we did not collect information from the patients we served regarding their HPV vaccination status. HPV vaccination is a method of preventing cervical cancer that is most effective administered to adolescents and young adults (ideally, those aged 11–12 years but up to age 26) who have had limited exposure to HPV.^21^ The first generation of the vaccine was first introduced in 2006, at which time it provided protection against 4 of the more than 40 known cancer-causing strains of HPV.^5^

However, it is the second generation of the vaccine, which was introduced in 2014 and protects against five additional strains of cervical cancer-causing HPV, which is widely used today.^5^ Given that nearly 15 years have passed since the HPV vaccine was first made available, it is likely that the effectiveness of the vaccine reduced the number of positive cervical cancer screening outcomes that we observed among the younger women served by our program. While we do not have information about our patients' vaccination statuses, and as a result, are not able to determine to what extent this may be true, we have several reasons to believe that our results and conclusions would not be significantly affected.

First, more than 90% of our sample of women were older than 30 years and thus were already older than the target age of vaccination when the vaccine was first introduced. Furthermore, even today, HPV vaccination rates remain particularly low (37%) among uninsured individuals within the target vaccination age groups.^5^ Most importantly, because we found evidence of a statistically significant inverse relationship between patient age and the positive Pap test outcomes, any additional positive tests that would have been observed in the absence of the vaccine would only make this effect more pronounced.

While we acknowledge the limitations of our sample, we feel that the strengths of this research outweigh its limitations. Cervical cancer screening outcomes for our sample of 841 women were sourced directly from medical records, rather than from a self-reported survey, which is subject to recall bias. In addition, our sample of low-income uninsured women represents a group that is not likely to be observed in large insurance claims-based studies. Thus, this study provides unique insight into the association between demographic factors and cervical cancer screening outcomes within a less accessible priority population group.

## Conclusions

Our study showed a decline in the rate of positive cytological outcomes with increased age, like that of histological trends. Our findings support recommendations of the U.S. Preventive Services Task Force to discontinue screening for women older than 65 years at low risk for cervical cancer.

While we did not find evidence of an association between cervical screening outcomes and the other sociodemographic characteristics we tested, this may be due in part to sample size constraints. While this study was performed using a relatively large sample of low-income and uninsured women (*n* = 841) who were served over a 6-year period, the limited number of positive findings present in the data created a statistical power problem. Our difficulty achieving the statistical power needed to perform additional statistical tests highlights that sample size constraints are a significant barrier to studying health outcomes within low-income and uninsured populations, which are often missing in large data sets (*e.g.*, claims databases) that are used in cross-sectional research.

## Abbreviations Used

ASCUS: atypical squamous cells of undetermined significance
CIN: cervical intraepithelial neoplasia
HGSIL: high-grade squamous intraepithelial lesion
HPV: human papillomavirus
LGSIL: low-grade squamous intraepithelial lesion
N/A: not applicable
Pap test: Papanicolaou test
RUCA: Rural–Urban Commuting Area
U/S: unsatisfactory sample
